# Exercise and Inflammation

**DOI:** 10.3390/antiox8060155

**Published:** 2019-06-02

**Authors:** Llion Roberts, Katsuhiko Suzuki

**Affiliations:** 1 School of Allied Health Sciences, Griffith University, Gold Coast 4215, Australia; 2 Faculty of Sport Sciences, Waseda University, Tokorozawa, Saitama 359-1192, Japan; katsu.suzu@waseda.jp

Exercise and inflammation induce multi-faceted physiological responses in their own right, let alone when considered together. For example, it is widely accepted that aerobic exercise augments numerous key regulatory parameters associated with health and disease. Clinically important transient increases in insulin sensitivity and glucose transporter translocation are observed in healthy individuals across the lifespan [1,2,3], and within diabetic individuals [4], whilst emerging evidence also supports such responses after resistance-type training [5]. Subsequently, a combined endurance and resistance approach to improve the control of glycemic and lipid profiles in diabetic individuals is promoted [6]. This example has led to a growing onus being paced upon exercise as a cornerstone for the prevention and treatment of lifestyle-related diseases such as diabetes. The development, existence, and function of inflammation, on the other hand, have been considered paradoxical in recent times related to wide-ranging pathological conditions from coronary artery diseases [7] to upper respiratory tract infections [8] and chronic obstructive pulmonary disease [9], and more inherently with ageing [10]. Further, on a fundamental level, our understanding is increasing regarding how inflammation contributes to regulating muscle homeostasis and myogenesis [11]. In an exercise context, this understanding is crucial, given the likely hermetic association with regulating adaptations to chronic exercise, and the association with ageing. Intriguingly, the suppression of inflammation via exogenous supplementation of cyclooxygenase inhibitors seems to attenuate adaptations in the young [12] but not the old [13]. Therefore, it is crucial for us to develop a greater understanding of how these factors are independently influenced by exercise, as well as how exercise regulates the interrelationship between the two, for example, by means of oxidative stress and redox control [12].

The aim of this Special Issue was to compile a series of both original investigations and review articles spanning these areas, to collectively contribute to the enhancement of our understanding in this area. Collectively, this Special Issue contains six review articles and six empirical investigations, spanning molecular mechanisms, nutritional and neutraceutical supplementation, and pathological manifestations. Review articles by Nemes and colleagues [14], and Suzuki [15], firstly provide holistic insights into the mechanisms involved with reactive oxygen species and reactive nitrogen species regulation of muscle contractions, as well as the exercise-induced influences upon cytokine dynamics, respectively. These reviews are complimented by overviews of the influences of specific and more general dietary factors, such as β-hydroxy-β-methylbutyrate (HMB) by Arazi and colleagues [16], and antioxidant intake by Kawamura and Muraoka [17]. Finally, perspectives relating to the pathophysiological conditions of atherosclerosis [18] and heat stroke [19] are provided. Empirical studies include an in vitro model of muscle cell damage [20], the investigation of nutrient supplementation such as alpha-lipoic acid [21], HMB [16], barley-wheat grass juice [22] and graded carbohydrate intake [23], and an investigation into the efficacy of post-exercise hydrogen baths [24].

From the empirical nutritional supplementation studies, a combination of acute pre- and acute post-exercise supplementation models are utilized, along with chronic supplementation alongside exercise training, and chronic supplementation alone. Georgakuli and colleagues [21] investigated how chronic alpha-lipoic acid supplementation alone may augment exercise and redox status in glucose-6-phosphate dehydrogenase-deficient individuals. Such deficiency subjects the individuals to compromised glutathione levels and increased susceptibility to oxidative stress; thus, such repercussions were investigated systemically from blood, in response to an acute exercise insult performed before and after the supplementation period. Resting markers of oxidative defense from total antioxidant capacity and bilirubin concentrations were increased after four weeks of supplementation, but such effects were absent post-exercise. 

Implementing a chronic supplementation regime alongside exercise training is a popular approach aimed at promoting adaptation in a number of contexts. Given the identification of leucine as a nutrient ‘trigger’ for muscle anabolism in recent years [25], it is unsurprising that its metabolite, HMB, is receiving much attention as a potential promoter of adaptation to resistance training. Arazi and colleagues [16] examined the effects of HMB free acid supplementation on resting systemic oxidative stress markers following six weeks of resistance training. Strength training induced reductions in oxidative stress, as identified by reductions in malondialdehyde concentrations, and a marker of protein carbonylation. However, no differences were identified following HMB supplementation.

A multiple (low and high) dose-response investigation in to the effects of an extended (7 day) dose of a daily plant-based nutraceutical, barley-wheat grass juice was conducted by Williamson and colleagues [22]. Acute resting peripheral cell mononuclear DNA damage was subsequently examined in response to the supplementation period, and post-exercise, after an acute bout of high-intensity exercise that followed each supplementation period. DNA damage increased in response to each exercise bout, irrespective of the preceding supplementation dose. Another study that investigated the acute multiple (low and high) dose-response effects of nutrient ingestion was conducted by Tanisawa and colleagues [23]. In this instance, the temporal responses of neutrophil activation and circulating cytokines were compared between low and high doses of carbohydrate consumption, after an endurance exercise task. Herein, they identified favorable conditions for exercise recovery following supplementation that were not associated with elevations in inflammatory responses.

Hydrogen baths are frequently used post-exercise, supported by anecdotal evidence of enhanced perceptions of recovery and wellbeing. Empirical evidence regarding this approach is lacking, however. Kawamura and colleagues [24] examined the influences of a hydrogen bath on muscle soreness and function, alongside neutrophil dynamics after muscle damage induced by a bout of downhill-running. Whilst circulating interleukin-6 concentrations were identified as being associated with neutrophil numbers and additional markers of muscle damage, there was no detectable effect of the hydrogen bath on physiological parameters or recovery. As such, the hydrogen bath did not offer any additional benefits over and above a placebo bath. An in vitro muscle cell damage model was utilized by Yano and colleagues [20], to investigate the cellular underpinnings of macrophage chemotaxis. By manipulating culture medium in the presence or absence of lipopolysaccharide combined with the phosphoinositide 3-kinase (PI3K) inhibitor, Ly294002, the role of PI3K in macrophage chemotaxis was investigated. Following the induction of muscle cell damage by liquid nitrogen and heat exposure, macrophage chemotaxis was identified as being dependent on PI3K/Protein Kinase B pathway activation.

Collectively, the use of exercise to promote health and prevent and improve disease states has become known as “Exercise is Medicine”, and is considered as a valuable non-pharmacological therapy in many international societies. However, exercise has the capacity to induce muscle damage and fatigue that may become stressors to the body, thus inducing acute inflammation and increasing susceptibility to infection [15,26]. As such, a better understanding of current biomarkers, and the identification and understanding of new candidate biomarkers are required to help reveal the effects of exercise from a pathological point of view, or to develop early prognostic markers [26,27,28]. However, future examination of the implementation and optimization of exercise approaches alone, and in combination with other efficacious influences such as diet and lifestyle factors are warranted, and are anticipated to become more actively promoted and highlighted in the future [29].
